# Synthesis and structure of clozapine *N*-oxide hemi(hydro­chloride): an infinite hydrogen-bonded poly[*n*]catenane

**DOI:** 10.1107/S2056989022009306

**Published:** 2022-09-27

**Authors:** Phillip L. van der Peet, Rohan D. Joyce, Holger Ott, Sebastian M. Marcuccio, Jonathan M. White, Spencer J. Williams

**Affiliations:** a School of Chemistry and Bio21 Molecular Science and Biotechnology Institute, University of Melbourne, Parkville, 3010, Australia; b Advanced Molecular Technologies, Unit 1, 7-11 Rocco Drive, Scoresby, Victoria, 3179, Australia; c Bruker AXS GmbH, Oestliche Rheinbrueckenstr. 49, 76187 Karlsruhe, Germany; University of Aberdeen, Scotland

**Keywords:** hydrogen bonding, crystal structure, hydrogen-bonding catenation

## Abstract

The structure of the title compound, 2C_18_H_19_ClN_4_O·HCl or (CNO)_2_·HCl (C_36_H_39_Cl_3_N_8_O_2_), at 100 K has tetra­gonal (*I*4/*m*) symmetry. The dihedral angle between the benzene rings of the fused ring system of the CNO mol­ecule is 40.08 (6)° and the equivalent angle between the seven-membered ring and its pendant *N*-oxide ring is 31.14 (7)°. The structure contains a very strong, symmetrical O—H⋯O hydrogen bond [O⋯O = 2.434 (2) Å] between two equivalent *R*_3_N^+^—O^−^ moieties, which share a proton lying on a crystallographic twofold rotation axis. These units then form a (CNO)_4_·(HCl)_2_ ring by way of two equivalent N—H⋯Cl hydrogen bonds (Cl^−^ site symmetry *m*). These rings are catenated into infinite chains propagating along the *c*-axis direction by way of shape complementarity and directional C—H⋯N and C—H⋯π inter­actions.

## Abstract 2

The recrystallization of clozapine *N*-oxide hydro­chloride from a range of solvents leads to the loss of half an equivalent of HCl and the formation of single crystals of a hydrogen-bond-linked poly[*n*]catenane of clozapine *N*-oxide hemi­hydro­chloride.

## 1. Chemical context

Coordination-driven self-assembly of supra­molecular structures is a major focus area of materials science. However, hydrogen-bond-driven self-assembly has been less well studied, most likely as a consequence of the weakness of hydrogen bonding relative to coordinate bonding. Nevertheless, the directionality of hydrogen bonding can lend it to the controllable formation of supra­molecular networks (González-Rodríguez & Schenning, 2011 ▸; Steiner, 2002 ▸; Prins *et al.*, 2001 ▸). The simplest infinite inter­locking systems are the one-dimensional polycatenanes (poly[*n*]catenanes). Such systems have been described involving inter­penetrating metallocycles of silver/bis­(2-methyl­imidazol­yl) (Jin *et al.*, 2006 ▸, 2008 ▸, 2018 ▸) and mercury/1,2-bis­[(pyridin-4-yl­thio)­meth­yl]benzene (Xue *et al.*, 2015 ▸). However, the lack of many examples beyond these suggests that the self-assembly of this inter­esting topological architecture is not easily achieved. Here, we report the serendipitous discovery of an infinite one-dimensional polycatenane architecture templated by a chloride anion that forms upon the attempted recrystallization of clozapine *N*-oxide (C_18_H_19_ClN_4_O; hereafter CNO) mono-hydro­chloride, an inactive metabolite of clozapine that is utilized as an actuator of engineered muscarinic acetyl­choline receptors (Armbruster *et al.*, 2007 ▸; Urban & Roth, 2015 ▸; Dong *et al.*, 2010 ▸; Gomez *et al.*, 2017 ▸).

As part of efforts to develop a water-soluble salt form of CNO (van der Peet *et al.*, 2018 ▸) we synthesized CNO·HBr and CNO·HCl by formation of the salt in methanol (Scheme 1). 
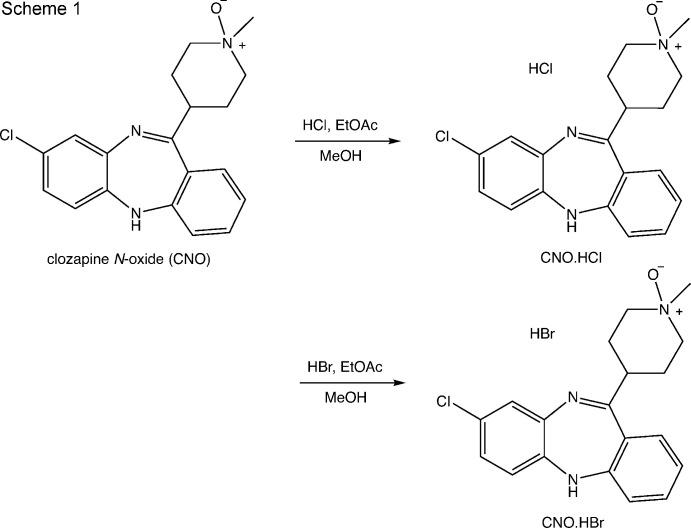


The latter compound has been reported previously (Allen *et al.*, 2019 ▸), but its preparation was not described. Elemental analysis of the precipitated CNO·HCl was consistent with the proposed structure in Scheme 1. Although crystals suitable for single crystal X-ray analysis were not obtained from the crude precipitate, powder X-ray diffraction of the precipitate suggested the material was substanti­ally crystalline. To obtain structural verification and to locate the site of protonation, we attempted to grow single crystals of CNO·HCl for single crystal X-ray analysis. Slow evaporation of a solution of CNO·HCl from a variety of solvents, or by diffusion of diethyl ether into a variety of solvents consistently yielded small orange block-shaped crystals of the title hemi­hydro­chloride, which were found to be no longer soluble in water or other solvents (Scheme 2). 
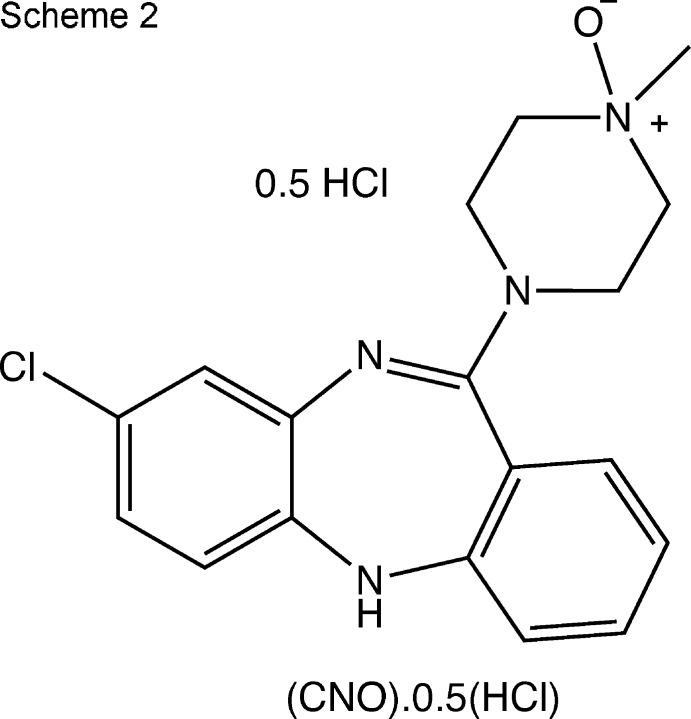


## 2. Structural commentary

**Figure 1:**
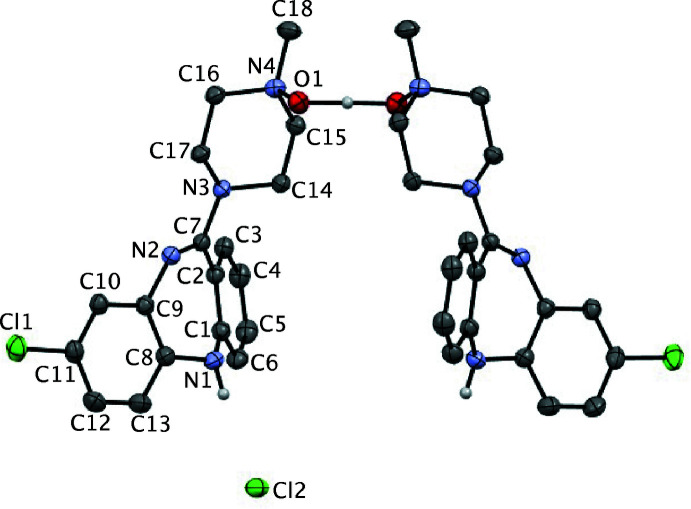
The mol­ecular structure of (CNO)_2_·HCl showing 50% displacement ellipsoids with C-bound H atoms omitted for clarity. The unlabelled atoms are generated by the symmetry operation −*x*, 1 − *y*, *z*.

**Figure 2:**
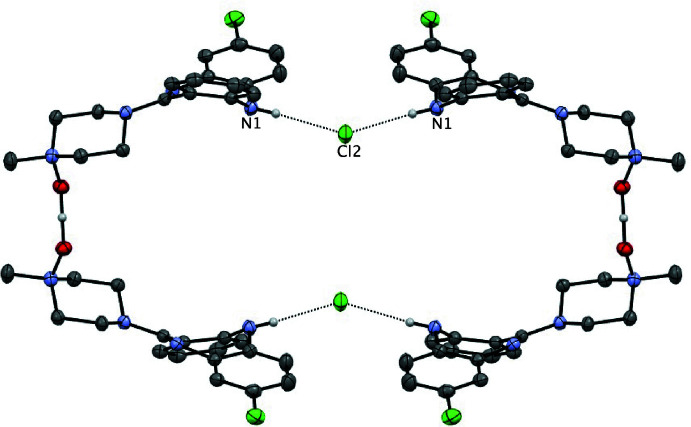
The cyclic tetra­mer (CNO)_4_·(HCl)2 templated by N—H⋯Cl hydrogen-bonding inter­actions.

Single-crystal X-ray diffraction analysis of the orange crystals revealed that the CNO·HCl salt implied by the analysis for the initially formed salt (above) had lost half an equivalent of HCl upon crystallization and crystallized as a hemi­hydro­chloride, (CNO)_2_·HCl, in the tetra­gonal space group *I*4/*m* (Scheme 2 and Fig. 1 ▸). In this structure, two mol­ecules of CNO, which are related by a crystallographic twofold axis, share a proton, which is located on the rotation axis and forms a strong, essentially linear and apparently symmetric O—H⋯O/O⋯H—O hydrogen bond between the two mol­ecules *via* the *N*-oxide moieties [O1⋯O1^i^ = 2.434 (2) Å; symmetry code (i) −*x*, 1 − *y*, *z*]. Within the structure, the chloride counter-ion (Cl2) is located on a crystallographic mirror plane and accepts equivalent N—H⋯Cl hydrogen bonds [N1⋯Cl2 = 3.3259 (14) Å] to two mirror-related (CNO)_2_H^+^ moieties resulting in the formation of a cyclic structure templated by the Cl^−^ counter-ions (Fig. 2 ▸). The diazepine ring core in (CNO)_2_·HCl adopts a boat conformation (Table 1 ▸) in which the N1(H) group is at the bow and the C7=N2 imine group is the stern. A consequence of the boat conformation is the mean planes of the two fused benzene rings lie at an angle 40.08 (6)° to one another; this represents a less puckered ring to that observed in the (CNO)·MeOH solvate in which the aromatic rings are at an angle of 56.2° (van der Peet *et al.*, 2018 ▸) demonstrating the flexibility of this ring system. The equivalent angle between the seven-membered diazepine ring and its pendant *N*-oxide ring is 31.14 (7)°

## 3. Supra­molecular features

**Figure 3:**
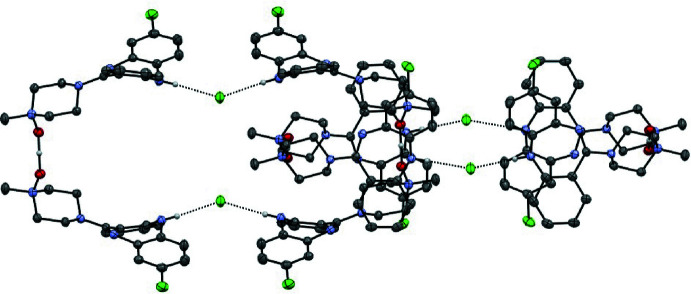
Partial structure of (CNO)_2_·HCl catenated chain showing two members of the poly[*n*]catenane; adjacent links in the chain are related by a 4_2_ screw axis.

**Figure 5:**
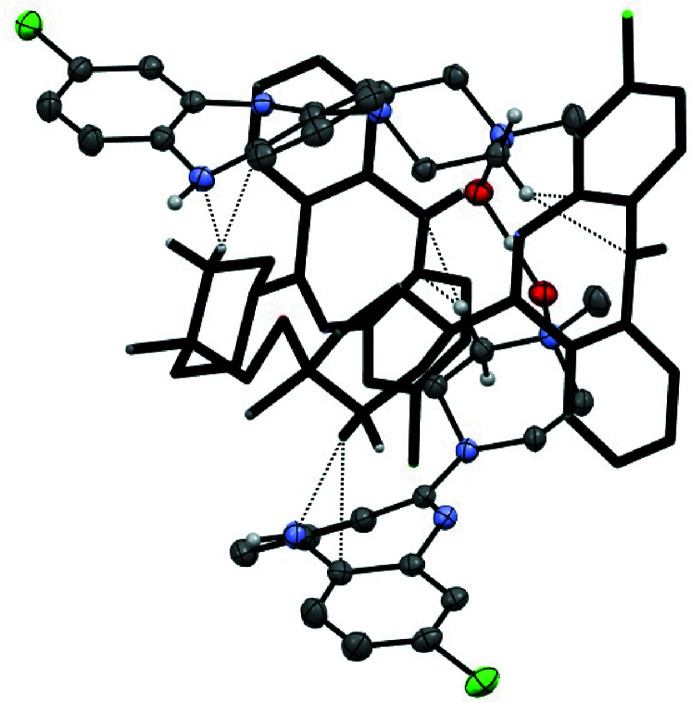
C—H⋯N and C—H⋯π inter­actions at the inter­face of neighbouring tetra­meric (CNO)_4_·(HCl) rings in the poly[*n*]catenane.

**Figure 6:**
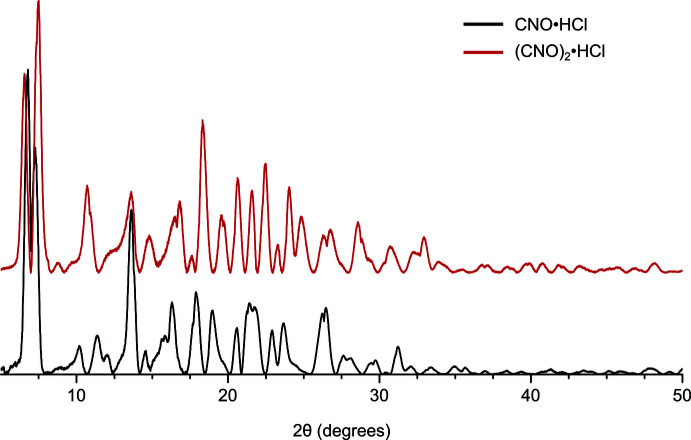
Overlay of powder patterns of the initial precipitate of (CNO)·HCl and the recrystallized material (CNO)_2_·HCl.

The tetra­meric cyclic structures are catenated and form infinite chains extending along the *z*-direction (Figs. 3 ▸ and 4 ▸) in which adjacent links in the chain are related by a 4_2_ screw axis. The catenated rings form both as a result of general complementarity in the shapes of the inter­nal cavities of the inter­acting (CNO)_2_ dimers related by the symmetry operation ( 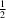 − *y*, 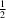 + *x*, 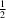 − *z*), and further stabilized by four equivalent non-classical hydrogen-bonding inter­actions involving the polarized C—H bond adjacent to the *N*-oxide moiety; (C15—H15*A*⋯N1, Table 2 ▸) in addition to four equivalent C—H⋯π inter­actions [H15*A*⋯C8 = 2.706 (2) Å] (Fig. 5 ▸). Solvent voids, which account for approximately 17% of the unit-cell volume, lie between the catenated chains: the disordered solvent was accounted for using the Squeeze procedure in *PLATON* (Spek, 2015 ▸). To establish the relationship between the original material and that obtained after crystallization, powder X-ray diffraction data were obtained for the orange crystals and compared to that for the original material (Fig. 6 ▸). The two powder diffraction patterns are substanti­ally different, which is consistent with the combustion analysis of the original material that analysed as (CNO)·HCl, whereas the crystallized material is (CNO)_2_·HCl. Application of the same approach to CNO·HBr did not lead to an equivalent polymeric material.

## 4. Database survey

The formation of strong hydrogen bonds is predicted to occur when the p*K*_a_ value for the donor acid matches that for the acceptor’s conjugate acid form (Gilli *et al.*, 2009 ▸). In this structure, a strong hydrogen bond between a protonated tertiary amine *N*-oxide and its conjugate base is predicted. A search of the Cambridge Structural Database (2022.2.0, September 2022; Groom *et al.*, 2016 ▸) for structures containing the *R*_3_N—OH⋯O—N*R*_3_ moiety with constraints on the *R* factor to 5% or less and only organic structures surveyed gave eight good-quality structures (CSD refcodes: RAJDAL (Bettencourt *et al.*, 2021 ▸), AJESEQ (Wlaźlak *et al.*, 2018 ▸), AREREW (Moore *et al.*, 2016 ▸), BAYDEK (Jaskólski *et al.*, 1982 ▸), EPSPOX (Małuszyńska & Okaya, 1977 ▸), FUBMAS (Moore *et al.*, 2015 ▸), NUCDUK (Krzywda *et al.*, 1996 ▸) and OBECUV (Bohmer *et al.*, 2011 ▸): these structures are characterized by O⋯O distances ranging from 2.426–2.445 Å, which is comparable to the O⋯O distance of 2.434 (2) Å in this structure, thus all can be classified as strong O—H⋯O hydrogen bonds as predicted.

## 5. Synthesis and crystallization

*Preparation of clozapine N-oxide hydro­chloride (CNO*·*HCl)*

A 250 ml round-bottom flask was charged with clozapine *N*-oxide (5.00 g, 0.015 mol) and methanol (50 ml) and stirred under N_2_. Initially, the solid dissolved but then precipitated as a presumed CNO·methano­late adduct. A solution of HCl in ethyl acetate (2.8 *M*, 6 ml, 0.017 mol, 1.1 eq) was added slowly to the suspension. After 10 min the solid dissolved, and then precipitated as a faint yellow solid. The suspension was stirred for 1 h, then the solid was collected by filtration, and washed with ethyl acetate to afford CNO·HCl as a yellow solid (2.2 g, 39%). Degradation point: 473–478 K (corrected); ^1^H NMR (400 MHz, CD_3_OD) δ 3.35–3.45 (*m*, 6 H), 3.65–3.80 (*m*, 4 H), 3.95 (*br s*, 2 H), 6.83 (*d*, *J* 8.4 Hz, 1 H), 6.87 (*d*, *J* 2.4, 8.4 Hz, 1 H), 6.97 (*dd*, *J* = 2.4 Hz, 1H), 7.01 (*dd*, *J* 1.0, 8.0 Hz, 1H), 7.06 (*dt*, *J* 1.1, 7.6 Hz, 1H), 7.33 (*dd*, *J* 1.4, 7.8 Hz, 1H), 7.37 (*dt*, *J* 1.5, 6.4 Hz, 1H); ^13^C NMR (100 MHz, CD_3_OD) δ 43.1, 58.9, 65.3, 121.5, 121.6, 123.6, 124.3, 125.1, 127.4, 129.6, 131.2, 133.9, 142.5, 143.1, 155.5, 164.0. Elemental analysis: calculated for C_18_H_20_Cl_2_N_4_O: C 57.0, H 5.3, N 14.8. Found: C 56.8, H 5.6, N 14.8.

*Preparation of clozapine N-oxide hemi­hydro­chloride (CNO)_2_·HCl*

The above material (CNO·HCl) was recrystallized by diffusion of diethyl ether into a methanol solution giving (CNO)_2_·HCl as small orange blocks.

*Preparation of clozapine N-oxide hydro­bromide*

A 25 ml round-bottom flask was charged with clozapine *N*-oxide (1.00 g, 2.92 mmol, 1 eq.) and methanol (5 ml) and stirred under N_2_. Initially, the solid dissolved but then precipitated as a presumed CNO·methano­late adduct. The solution was cooled in an ice–water bath and 48% HBr in water (0.35 ml, 3.07 mmol, 1.05 eq) was added slowly to the suspension. The mixture stirred for 1 h at rt, without formation of a precipitate. The solvent was evaporated and the residue suspended in EtOAc (10 ml). The resulting solid was collected by filtration and washed with EtOAc to afford CNO·HBr as a yellow solid (1.1 g, 89%). Degradation point: 483–493 K (corrected); ^1^H NMR (400 MHz, CD_3_OD) δ 3.68 (*s*, 3 H), 3.87 (*br d*, *J* 11.6 Hz, 2 H), 3.9–4.2 (*m*, 6 H), 7.01 (*d*, *J* 8.6 Hz, 1 H), 7.13–7.23 (*m*, 3 H), 7.27 (*br s*, 1 H), 7.53–7.60 (*m*, 2 H); ^13^C NMR (100 MHz, CD_3_OD) δ 44.7, 57.8, 57.9, 64.6, 64.7, 122.6, 124.9, 125.0, 126.7, 126.8, 128.4, 130.0, 133.1, 136.5, 145.9, 156.4.

## 6. Refinement

Crystal data, data collection and structure refinement details are summarized in Table 3 ▸. Regions of the unit cell occupied by disordered solvent (1409 Å^3^; ≃ 18.1% of the unit-cell volume) were processed with the Squeeze algorithm in *PLATON* (Spek, 2015 ▸); the stated composition, density, *etc*. do not take account of the solvent.

## Supplementary Material

Crystal structure: contains datablock(s) I. DOI: 10.1107/S2056989022009306/hb8039sup1.cif

Structure factors: contains datablock(s) I. DOI: 10.1107/S2056989022009306/hb8039Isup2.hkl

CCDC reference: 2208459

Additional supporting information:  crystallographic information; 3D view; checkCIF report

## Figures and Tables

**Figure 4:**
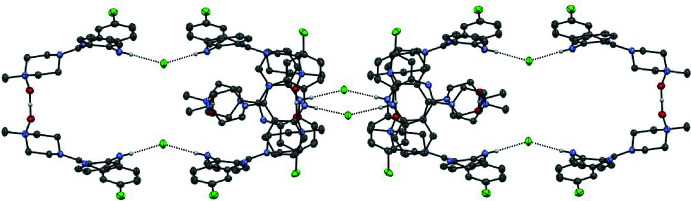
Partial structure of (CNO)_2_·HCl catenated chain showing three members of the poly[*n*]catenane; adjacent links in the chain are related by a 4_2_ screw axis.

**Table 1:** Selected torsion angles (°)

C8—N1—C1—C2	55.7 (2)	C7—N2—C9—C8	33.5 (2)
C9—N2—C7—C2	3.2 (2)	C1—N1—C8—C9	−54.0 (2)
N1—C1—C2—C7	0.9 (2)	N2—C9—C8—N1	−4.9 (2)
N2—C7—C2—C1	−35.2 (2)		

**Table 2:** Hydrogen-bond geometry (Å, °)

*D*—H⋯*A*	*D*—H	H⋯*A*	*D*⋯*A*	*D*—H⋯*A*
O1—H1*A*⋯O1^i^	1.22 (1)	1.22 (1)	2.434 (2)	179 (4)
N1—H1⋯Cl2	0.86 (2)	2.46 (2)	3.3259 (14)	176 (2)
C15—H15*A*⋯O1^i^	0.97	2.64	3.2598 (19)	122
C15—H15*A*⋯N1^ii^	0.97	2.51	3.416 (2)	156
C15—H15*B*⋯Cl1^iii^	0.97	2.91	3.8431 (17)	162

Symmetry codes: (i) 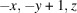 ; (ii) 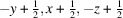 ; (iii) 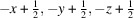 .

**Table 3:** Experimental details

Crystal data	
Chemical formula	2C_18_H_19_ClN_4_O·HCl
*M*_r_	722.10
Crystal system, space group	Tetragonal, *I*4/*m*
Temperature (K)	100
*a*, *c* (Å)	17.305 (2), 26.040 (5)
*V* (Å^3^)	7798 (3)
*Z*	8
Radiation type	Synchrotron, λ = 0.710757 Å
μ (mm^−1^)	0.28
Crystal size (mm)	0.06 × 0.05 × 0.04

Data collection	
Diffractometer	ADSC Quantum 210r
No. of measured, independent and observed [*I* > 2σ(*I*)] reflections	66433, 5901, 5096
*R*_int_	0.049
(sin θ/λ)_max_ (Å^−1^)	0.741

Refinement	
*R*[*F* ^2^ > 2σ(*F* ^2^)], *wR*(*F* ^2^), *S*	0.049, 0.138, 1.03
No. of reflections	5901
No. of parameters	231
H-atom treatment	H atoms treated by a mixture of independent and constrained refinement
Δρ_max_, Δρ_min_ (e Å^−3^)	0.81, −0.49

Computer programs: AS QEGUI, *XDS* (Kabsch, 1993 ▸), *SHELXT* (Sheldrick, 2015*a* ▸), *SHELXL2016/6* (Sheldrick, 2015*b* ▸), *Mercury* (Macrae *et al.*, 2020 ▸) and *WinGX* (Farrugia, 2012 ▸).

## supplementary crystallographic information

### Crystal data

**Table:**

2C_18_H_19_ClN_4_O·HCl	*D*_x_ = 1.230 Mg m^−^^3^
*M**_r_* = 722.10	Synchrotron radiation, λ = 0.710757 Å
Tetragonal, *I*4/*m*	Cell parameters from 5908 reflections
*a* = 17.305 (2) Å	θ = 1.4–31.8°
*c* = 26.040 (5) Å	µ = 0.28 mm^−^^1^
*V* = 7798 (3) Å^3^	*T* = 100 K
*Z* = 8	Block, yellow
*F*(000) = 3024	0.06 × 0.05 × 0.04 mm

### Data collection

**Table:**

ADSC Quantum 210r diffractometer	5096 reflections with *I* > 2σ(*I*)
Radiation source: MX1 Beamline Australian Synchrotron	*R*_int_ = 0.049
Silicon Double Crystal monochromator	θ_max_ = 31.8°, θ_min_ = 1.4°
ω Scan scans	*h* = −25→25
66433 measured reflections	*k* = −25→25
5901 independent reflections	*l* = −36→36

### Refinement

**Table:**

Refinement on *F*^2^	Hydrogen site location: mixed
Least-squares matrix: full	H atoms treated by a mixture of independent and constrained refinement
*R*[*F*^2^ > 2σ(*F*^2^)] = 0.049	*w* = 1/[σ^2^(*F*_o_^2^) + (0.0661*P*)^2^ + 11.4576*P*] where *P* = (*F*_o_^2^ + 2*F*_c_^2^)/3
*wR*(*F*^2^) = 0.138	(Δ/σ)_max_ = 0.001
*S* = 1.03	Δρ_max_ = 0.81 e Å^−^^3^
5901 reflections	Δρ_min_ = −0.49 e Å^−^^3^
231 parameters	Extinction correction: SHELXL2016/6 (Sheldrick 2015b), Fc^*^=kFc[1+0.001xFc^2^λ^3^/sin(2θ)]^-1/4^
0 restraints	Extinction coefficient: 0.0069 (5)

### Special details

**Table:**

Geometry. All esds (except the esd in the dihedral angle between two l.s. planes) are estimated using the full covariance matrix. The cell esds are taken into account individually in the estimation of esds in distances, angles and torsion angles; correlations between esds in cell parameters are only used when they are defined by crystal symmetry. An approximate (isotropic) treatment of cell esds is used for estimating esds involving l.s. planes.

### Fractional atomic coordinates and isotropic or equivalent isotropic displacement parameters (Å^2^)

**Table:**

	*x*	*y*	*z*	*U*_iso_*/*U*_eq_	
Cl1	0.13932 (3)	0.04426 (2)	0.37292 (2)	0.03680 (13)	
Cl2	0.16534 (4)	0.44691 (3)	0.500000	0.03563 (15)	
O1	0.02559 (6)	0.43450 (7)	0.13599 (5)	0.0285 (2)	
N3	0.17304 (7)	0.39022 (7)	0.21729 (5)	0.0225 (2)	
N2	0.15846 (7)	0.30145 (7)	0.28157 (5)	0.0228 (2)	
N1	0.18161 (8)	0.38441 (8)	0.37970 (5)	0.0261 (3)	
N4	0.10378 (8)	0.43810 (8)	0.12165 (5)	0.0248 (3)	
C1	0.23713 (9)	0.42470 (8)	0.35074 (6)	0.0242 (3)	
C7	0.18814 (8)	0.36630 (8)	0.26780 (5)	0.0216 (3)	
C2	0.24220 (8)	0.41696 (8)	0.29712 (6)	0.0228 (3)	
C10	0.15645 (9)	0.18517 (9)	0.32919 (6)	0.0252 (3)	
H10	0.150809	0.159189	0.298148	0.030*	
C14	0.14157 (9)	0.46872 (8)	0.21112 (6)	0.0239 (3)	
H14A	0.170572	0.504443	0.232387	0.029*	
H14B	0.088144	0.469610	0.222369	0.029*	
C9	0.16811 (8)	0.26540 (8)	0.32932 (6)	0.0228 (3)	
C16	0.13708 (9)	0.35868 (9)	0.12884 (6)	0.0254 (3)	
H16A	0.190585	0.358444	0.117741	0.030*	
H16B	0.108830	0.322118	0.107741	0.030*	
C15	0.14617 (9)	0.49399 (8)	0.15559 (6)	0.0245 (3)	
H15A	0.123725	0.545067	0.152003	0.029*	
H15B	0.199875	0.496810	0.145089	0.029*	
C3	0.29823 (9)	0.46033 (9)	0.27067 (6)	0.0256 (3)	
H3	0.302804	0.454729	0.235282	0.031*	
C11	0.15328 (9)	0.14442 (9)	0.37477 (6)	0.0291 (3)	
C5	0.33991 (10)	0.52005 (10)	0.34901 (7)	0.0317 (3)	
H5	0.371497	0.554918	0.366272	0.038*	
C8	0.17647 (9)	0.30343 (9)	0.37683 (6)	0.0253 (3)	
C17	0.13260 (9)	0.33393 (8)	0.18470 (5)	0.0237 (3)	
H17A	0.078931	0.330314	0.195175	0.028*	
H17B	0.156037	0.283374	0.188721	0.028*	
C6	0.28604 (9)	0.47695 (10)	0.37606 (6)	0.0291 (3)	
H6	0.282259	0.482756	0.411475	0.035*	
C4	0.34685 (9)	0.51128 (9)	0.29616 (7)	0.0294 (3)	
H4	0.383731	0.539293	0.278039	0.035*	
C13	0.17144 (12)	0.26047 (10)	0.42216 (6)	0.0352 (4)	
H13	0.176053	0.285737	0.453531	0.042*	
C12	0.15973 (11)	0.18114 (10)	0.42162 (7)	0.0361 (4)	
H12	0.156323	0.153376	0.452117	0.043*	
C18	0.10927 (11)	0.46237 (11)	0.06669 (6)	0.0345 (4)	
H18A	0.079973	0.427372	0.045739	0.052*	
H18B	0.162386	0.461630	0.056060	0.052*	
H18C	0.088990	0.513702	0.062973	0.052*	
H1	0.1789 (13)	0.3988 (13)	0.4114 (9)	0.037 (6)*	
H1A	0.000000	0.500000	0.1357 (17)	0.077 (14)*	

### Atomic displacement parameters (Å^2^)

**Table:**

	*U*^11^	*U*^22^	*U*^33^	*U*^12^	*U*^13^	*U*^23^
Cl1	0.0421 (2)	0.02417 (19)	0.0442 (2)	0.00136 (15)	0.01026 (17)	0.00663 (15)
Cl2	0.0513 (4)	0.0331 (3)	0.0224 (2)	0.0004 (2)	0.000	0.000
O1	0.0225 (5)	0.0313 (6)	0.0318 (6)	−0.0003 (4)	−0.0012 (4)	0.0007 (4)
N3	0.0265 (6)	0.0200 (5)	0.0210 (5)	−0.0002 (4)	−0.0015 (4)	−0.0017 (4)
N2	0.0234 (6)	0.0225 (5)	0.0224 (5)	0.0017 (4)	0.0001 (4)	−0.0013 (4)
N1	0.0321 (7)	0.0252 (6)	0.0211 (6)	0.0006 (5)	0.0007 (5)	−0.0034 (4)
N4	0.0263 (6)	0.0271 (6)	0.0210 (6)	−0.0012 (5)	0.0007 (4)	−0.0003 (4)
C1	0.0234 (6)	0.0237 (6)	0.0256 (7)	0.0024 (5)	−0.0024 (5)	−0.0013 (5)
C7	0.0216 (6)	0.0219 (6)	0.0214 (6)	0.0025 (5)	0.0000 (5)	−0.0024 (5)
C2	0.0219 (6)	0.0214 (6)	0.0249 (6)	0.0018 (5)	−0.0018 (5)	−0.0015 (5)
C10	0.0234 (6)	0.0241 (7)	0.0282 (7)	0.0020 (5)	0.0025 (5)	0.0007 (5)
C14	0.0272 (7)	0.0211 (6)	0.0233 (6)	0.0015 (5)	−0.0014 (5)	−0.0014 (5)
C9	0.0203 (6)	0.0236 (6)	0.0246 (6)	0.0021 (5)	0.0006 (5)	0.0006 (5)
C16	0.0286 (7)	0.0256 (7)	0.0220 (6)	−0.0001 (6)	0.0009 (5)	−0.0028 (5)
C15	0.0264 (7)	0.0228 (6)	0.0241 (6)	−0.0020 (5)	−0.0011 (5)	0.0003 (5)
C3	0.0239 (6)	0.0236 (6)	0.0293 (7)	0.0010 (5)	0.0000 (5)	−0.0010 (5)
C11	0.0286 (7)	0.0238 (7)	0.0349 (8)	0.0030 (6)	0.0051 (6)	0.0046 (6)
C5	0.0275 (7)	0.0284 (7)	0.0392 (9)	−0.0019 (6)	−0.0071 (6)	−0.0053 (6)
C8	0.0258 (7)	0.0257 (7)	0.0245 (7)	0.0022 (5)	−0.0009 (5)	0.0001 (5)
C17	0.0275 (7)	0.0230 (6)	0.0205 (6)	−0.0010 (5)	−0.0005 (5)	−0.0027 (5)
C6	0.0294 (7)	0.0304 (7)	0.0274 (7)	0.0016 (6)	−0.0056 (6)	−0.0055 (6)
C4	0.0233 (7)	0.0262 (7)	0.0387 (8)	−0.0019 (5)	−0.0006 (6)	−0.0014 (6)
C13	0.0494 (10)	0.0340 (8)	0.0224 (7)	0.0029 (7)	−0.0001 (7)	0.0013 (6)
C12	0.0477 (10)	0.0327 (8)	0.0279 (8)	0.0034 (7)	0.0032 (7)	0.0070 (6)
C18	0.0464 (9)	0.0368 (8)	0.0203 (7)	−0.0016 (7)	0.0000 (6)	0.0033 (6)

### Geometric parameters (Å, º)

**Table:**

Cl1—C11	1.7506 (17)	C9—C8	1.409 (2)
O1—N4	1.4051 (17)	C16—C17	1.518 (2)
O1—H1A	1.2169 (12)	C16—H16A	0.9700
N3—C7	1.4035 (18)	C16—H16B	0.9700
N3—C17	1.4692 (18)	C15—H15A	0.9700
N3—C14	1.4723 (18)	C15—H15B	0.9700
N2—C7	1.2852 (19)	C3—C4	1.388 (2)
N2—C9	1.4012 (19)	C3—H3	0.9300
N1—C1	1.406 (2)	C11—C12	1.380 (2)
N1—C8	1.406 (2)	C5—C6	1.386 (2)
N1—H1	0.86 (2)	C5—C4	1.390 (2)
N4—C18	1.494 (2)	C5—H5	0.9300
N4—C15	1.5016 (19)	C8—C13	1.398 (2)
N4—C16	1.502 (2)	C17—H17A	0.9700
C1—C6	1.403 (2)	C17—H17B	0.9700
C1—C2	1.405 (2)	C6—H6	0.9300
C7—C2	1.492 (2)	C4—H4	0.9300
C2—C3	1.406 (2)	C13—C12	1.388 (3)
C10—C11	1.382 (2)	C13—H13	0.9300
C10—C9	1.403 (2)	C12—H12	0.9300
C10—H10	0.9300	C18—H18A	0.9600
C14—C15	1.513 (2)	C18—H18B	0.9600
C14—H14A	0.9700	C18—H18C	0.9600
C14—H14B	0.9700		
			
N4—O1—H1A	107.9 (6)	N4—C15—H15A	109.5
C7—N3—C17	115.75 (12)	C14—C15—H15A	109.5
C7—N3—C14	116.31 (11)	N4—C15—H15B	109.5
C17—N3—C14	111.87 (11)	C14—C15—H15B	109.5
C7—N2—C9	126.07 (13)	H15A—C15—H15B	108.1
C1—N1—C8	120.58 (13)	C4—C3—C2	121.53 (15)
C1—N1—H1	114.0 (15)	C4—C3—H3	119.2
C8—N1—H1	109.5 (15)	C2—C3—H3	119.2
O1—N4—C18	109.17 (12)	C12—C11—C10	121.41 (15)
O1—N4—C15	110.03 (11)	C12—C11—Cl1	119.43 (13)
C18—N4—C15	110.59 (12)	C10—C11—Cl1	119.16 (13)
O1—N4—C16	107.20 (11)	C6—C5—C4	120.17 (15)
C18—N4—C16	110.61 (12)	C6—C5—H5	119.9
C15—N4—C16	109.18 (12)	C4—C5—H5	119.9
C6—C1—C2	119.37 (14)	C13—C8—N1	119.28 (14)
C6—C1—N1	118.67 (14)	C13—C8—C9	119.13 (15)
C2—C1—N1	121.88 (13)	N1—C8—C9	121.24 (13)
N2—C7—N3	116.40 (13)	N3—C17—C16	110.00 (12)
N2—C7—C2	128.37 (13)	N3—C17—H17A	109.7
N3—C7—C2	115.03 (12)	C16—C17—H17A	109.7
C1—C2—C3	118.60 (14)	N3—C17—H17B	109.7
C1—C2—C7	121.68 (13)	C16—C17—H17B	109.7
C3—C2—C7	119.68 (13)	H17A—C17—H17B	108.2
C11—C10—C9	120.58 (14)	C5—C6—C1	120.92 (15)
C11—C10—H10	119.7	C5—C6—H6	119.5
C9—C10—H10	119.7	C1—C6—H6	119.5
N3—C14—C15	110.58 (12)	C3—C4—C5	119.38 (15)
N3—C14—H14A	109.5	C3—C4—H4	120.3
C15—C14—H14A	109.5	C5—C4—H4	120.3
N3—C14—H14B	109.5	C12—C13—C8	121.79 (16)
C15—C14—H14B	109.5	C12—C13—H13	119.1
H14A—C14—H14B	108.1	C8—C13—H13	119.1
N2—C9—C10	114.92 (13)	C11—C12—C13	118.44 (15)
N2—C9—C8	125.70 (13)	C11—C12—H12	120.8
C10—C9—C8	118.63 (13)	C13—C12—H12	120.8
N4—C16—C17	110.97 (12)	N4—C18—H18A	109.5
N4—C16—H16A	109.4	N4—C18—H18B	109.5
C17—C16—H16A	109.4	H18A—C18—H18B	109.5
N4—C16—H16B	109.4	N4—C18—H18C	109.5
C17—C16—H16B	109.4	H18A—C18—H18C	109.5
H16A—C16—H16B	108.0	H18B—C18—H18C	109.5
N4—C15—C14	110.53 (12)		
			
C8—N1—C1—C6	−127.64 (15)	C18—N4—C15—C14	−179.16 (13)
C8—N1—C1—C2	55.7 (2)	C16—N4—C15—C14	−57.23 (15)
C9—N2—C7—N3	177.72 (13)	N3—C14—C15—N4	57.38 (16)
C9—N2—C7—C2	3.2 (2)	C1—C2—C3—C4	−1.4 (2)
C17—N3—C7—N2	−6.69 (19)	C7—C2—C3—C4	176.48 (14)
C14—N3—C7—N2	127.72 (14)	C9—C10—C11—C12	1.4 (2)
C17—N3—C7—C2	168.54 (12)	C9—C10—C11—Cl1	−179.55 (11)
C14—N3—C7—C2	−57.05 (17)	C1—N1—C8—C13	132.81 (16)
C6—C1—C2—C3	2.0 (2)	C1—N1—C8—C9	−54.0 (2)
N1—C1—C2—C3	178.72 (13)	N2—C9—C8—C13	168.37 (15)
C6—C1—C2—C7	−175.82 (13)	C10—C9—C8—C13	−1.2 (2)
N1—C1—C2—C7	0.9 (2)	N2—C9—C8—N1	−4.9 (2)
N2—C7—C2—C1	−35.2 (2)	C10—C9—C8—N1	−174.45 (14)
N3—C7—C2—C1	150.21 (14)	C7—N3—C17—C16	−166.50 (12)
N2—C7—C2—C3	146.92 (15)	C14—N3—C17—C16	57.13 (16)
N3—C7—C2—C3	−27.63 (19)	N4—C16—C17—N3	−57.28 (16)
C7—N3—C14—C15	166.35 (12)	C4—C5—C6—C1	−0.8 (2)
C17—N3—C14—C15	−57.55 (16)	C2—C1—C6—C5	−1.0 (2)
C7—N2—C9—C10	−156.61 (14)	N1—C1—C6—C5	−177.76 (15)
C7—N2—C9—C8	33.5 (2)	C2—C3—C4—C5	−0.3 (2)
C11—C10—C9—N2	−170.69 (14)	C6—C5—C4—C3	1.4 (2)
C11—C10—C9—C8	0.0 (2)	N1—C8—C13—C12	174.49 (17)
O1—N4—C16—C17	−61.75 (15)	C9—C8—C13—C12	1.1 (3)
C18—N4—C16—C17	179.32 (13)	C10—C11—C12—C13	−1.5 (3)
C15—N4—C16—C17	57.41 (16)	Cl1—C11—C12—C13	179.46 (14)
O1—N4—C15—C14	60.16 (15)	C8—C13—C12—C11	0.2 (3)

### Hydrogen-bond geometry (Å, º)

**Table:**

*D*—H···*A*	*D*—H	H···*A*	*D*···*A*	*D*—H···*A*
O1—H1*A*···O1^i^	1.22 (1)	1.22 (1)	2.434 (2)	179 (4)
N1—H1···Cl2	0.86 (2)	2.46 (2)	3.3259 (14)	176 (2)
C15—H15*A*···O1^i^	0.97	2.64	3.2598 (19)	122
C15—H15*A*···N1^ii^	0.97	2.51	3.416 (2)	156
C15—H15*B*···Cl1^iii^	0.97	2.91	3.8431 (17)	162

Symmetry codes: (i) −*x*, −*y*+1, *z*; (ii) −*y*+1/2, *x*+1/2, −*z*+1/2; (iii) −*x*+1/2, −*y*+1/2, −*z*+1/2.
